# Gallbladder Cancer with Human Chorionic Gonadotropin-Positive Tumor Cells: A Case Report and Review of the Literature

**DOI:** 10.70352/scrj.cr.26-0227

**Published:** 2026-09-10

**Authors:** Shinya Sakamoto, Motoyasu Tabuchi, Teppei Tokumaru, Sunao Uemura, Shuta Tamura, Rika Yoshimatsu, Manabu Matsumoto, Jun Iwata, Takehiro Okabayashi

**Affiliations:** 1 Department of Gastroenterological Surgery, Kochi Health Sciences Center, Kochi, Kochi, Japan; 2 Department of Radiology, Kochi Health Sciences Center, Kochi, Kochi, Japan; 3 Department of Diagnostic Pathology, Kochi Health Sciences Center, Kochi, Kochi, Japan

**Keywords:** gallbladder cancer, human chorionic gonadotropin, HCG-positive tumor

## Abstract

**INTRODUCTION:**

Human chorionic gonadotropin (HCG)–positive gallbladder cancer (GBC) is extremely rare and is associated with aggressive biological behavior and a poor prognosis.

**CASE PRESENTATION:**

An 84-year-old woman presented with locally advanced GBC with right hepatic artery encasement. After 2 courses of gemcitabine, cisplatin, and durvalumab therapy, tumor progression was controlled, and radical surgery was planned. The patient underwent an extended cholecystectomy with adjacent liver resection, extrahepatic bile duct resection, and biliary reconstruction via Roux-en-Y hepaticojejunostomy. Histological examination revealed adenocarcinoma containing HCG-positive tumor cells, and the pathological response to chemotherapy was poor. The patient developed a recurrence 9 months after surgery. Although chemotherapy was reintroduced after recurrence, the disease showed a limited response, and the patient eventually died 20 months after the initial diagnosis.

**CONCLUSIONS:**

HCG-positive GBC is a rare and aggressive disease. Although surgical resection may be feasible in selected cases, the prognosis remains poor.

## Abbreviations

ASC
: adenosquamous carcinoma
CC
: choriocarcinoma
CEA
: carcinoembryonic antigen
ETT
: epithelioid trophoblastic tumor
GBC
: gallbladder cancer
GCD
: gemcitabine, cisplatin, and durvalumab
GCP
: gemcitabine, cisplatin, and pembrolizumab
HCG
: human chorionic gonadotropin
ICI
: immune checkpoint inhibitor
PTBD
: percutaneous transhepatic biliary drainage
RHA
: right hepatic artery

## INTRODUCTION

HCG is most commonly produced during pregnancy or in uterine tumors and is secreted by syncytiotrophoblast cells of the placenta.^1)^ HCG-related carcinoma of the gallbladder is extremely rare and is characterized by rapid growth, widespread metastasis, and aggressive invasion of surrounding tissues.^2–4)^ The prognosis is generally poor because of the highly malignant nature of the tumor and the lack of an established treatment strategy.^5,6)^

Herein, we present a case of GBC with HCG-positive tumor cells that was managed surgically. Furthermore, we describe the pathological and clinical characteristics of HCG-associated GBC, including its prognosis.

## CASE PRESENTATION

**Fig. 1:**
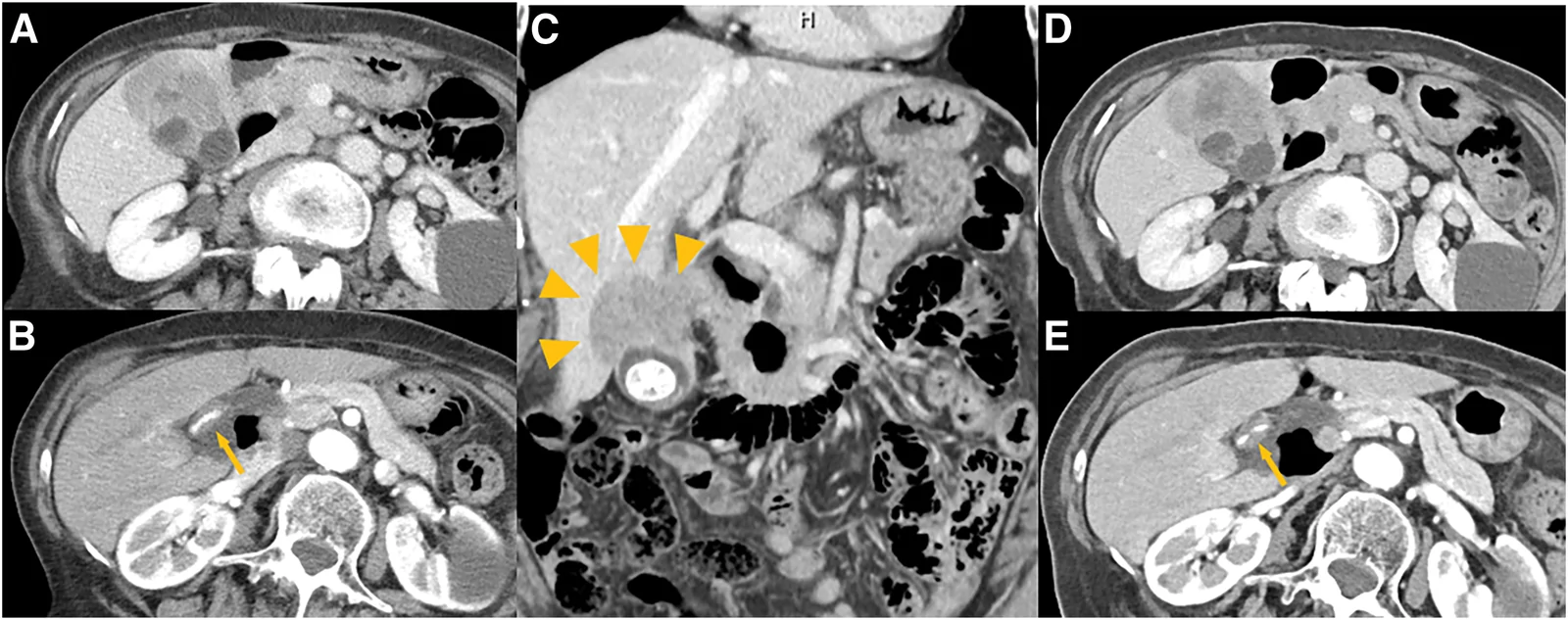
(**A**–**C**) Contrast-enhanced abdominal CT demonstrated thickening of the gallbladder fundus wall. The RHA appeared to be encased by enlarged hilar lymph nodes (arrow). The gallbladder tumor had invaded the adjacent liver parenchyma (arrowheds). (**D**, **E**) After 2 courses of GCD therapy, CT revealed that the primary lesion remained stable and that the RHA encasement had resolved (arrow). GCD, gemcitabine, cisplatin, and durvalumab; RHA, right hepatic artery

An 84-year-old woman was referred to our institute for evaluation of a gallbladder tumor detected on abdominal ultrasonography. Her medical history included essential hypertension, and she had undergone right total hip arthroplasty for osteoarthritis. Laboratory investigations revealed no remarkable abnormalities, except for a mildly elevated alanine aminotransferase level (116 U/L). The serum CEA level was elevated (60.8 ng/mL), whereas the serum carbohydrate antigen 19-9 level was within the normal range (9.2 U/mL). Contrast-enhanced abdominal CT revealed thickening of the gallbladder fundus wall. The gallbladder mass had invaded the adjacent liver parenchyma. The RHA was surrounded by enlarged hilar lymph nodes (**Fig. 1A**–**1C**). The tumor was suspected to involve the RHA via metastatic hilar lymph nodes, and curative resection would have required a right hepatectomy combined with resection of the inferior portion of segment 4. Because markedly elevated tumor marker levels may predict early recurrence, upfront surgery was considered less appropriate. Furthermore, considering the patient’s advanced age, postoperative morbidity was also a concern. Therefore, systemic chemotherapy was initiated prior to surgical intervention.

GCD therapy was initiated. After 2 courses of GCD therapy, the primary lesion remained stable. Although the tumor remained in contact with the RHA, there was no evidence of persistent encasement (**Fig. 1D** and **1E**). The primary lesion was controlled, no new metastases were identified, and RHA preservation was considered feasible. The serum CEA level (59.9 ng/mL) was slightly lower than that at the initial presentation. After the indication for surgery was carefully discussed, radical surgery for GBC was planned.

**Fig. 2:**
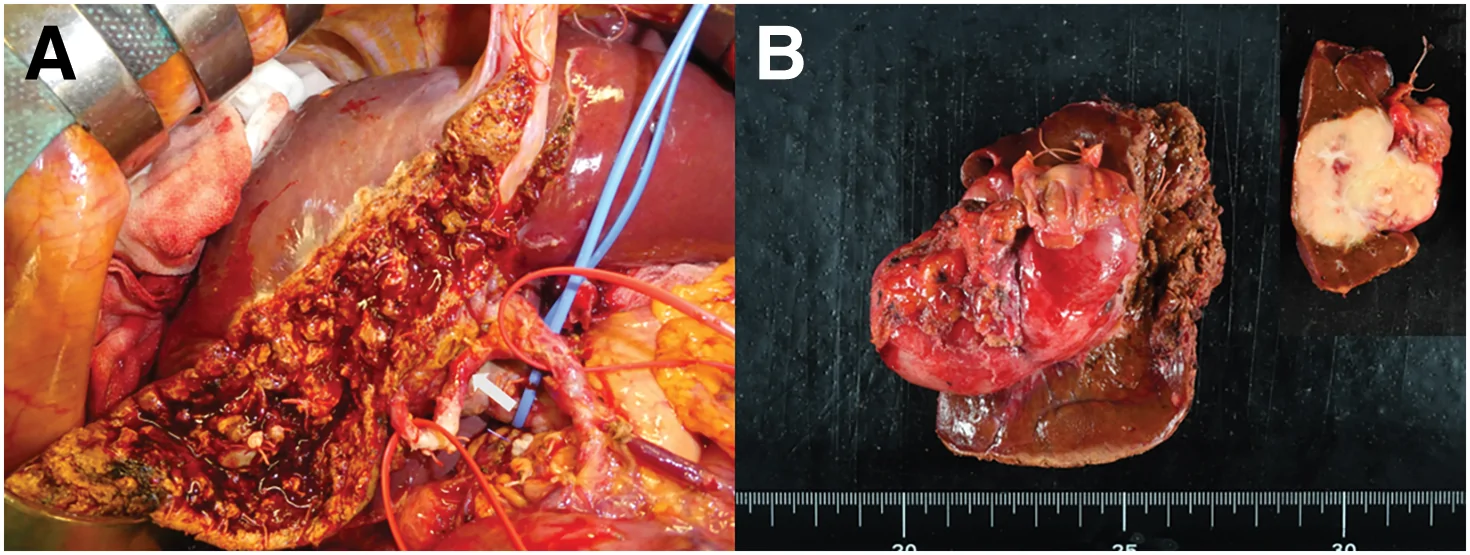
(**A**) Intraoperative findings. An extended cholecystectomy with adjacent liver resection was performed. Extrahepatic bile duct resection was also carried out, followed by biliary reconstruction using Roux-en-Y hepaticojejunostomy. The RHA was surrounded by a firm lymph node but was preserved (arrow). (**B**) Resected specimen. The lesion was removed en bloc, and the tumor had invaded the liver. RHA, right hepatic artery

Exploratory laparotomy revealed no peritoneal metastases. Intraoperative ultrasonography revealed no evidence of liver metastasis. The gallbladder tumor was in close contact with the duodenum, suggesting duodenal invasion. An extended cholecystectomy was performed with concomitant extrahepatic bile duct resection and partial resection of the duodenal wall. No direct tumor invasion of the RHA was identified. Although metastatic lymph nodes at the hepatic hilum were adjacent to the RHA, careful dissection allowed preservation of the vessel (**Fig. 2**). Biliary reconstruction was performed using Roux-en-Y hepaticojejunostomy. The duodenal wall defect was repaired by anastomosing the blind end of the jejunal limb.

Liver infarction of the remnant segment 5 and a liver abscess due to RHA occlusion were observed on POD 14. These complications improved with antibiotic therapy, and the patient was discharged on POD 24.

**Fig. 3:**
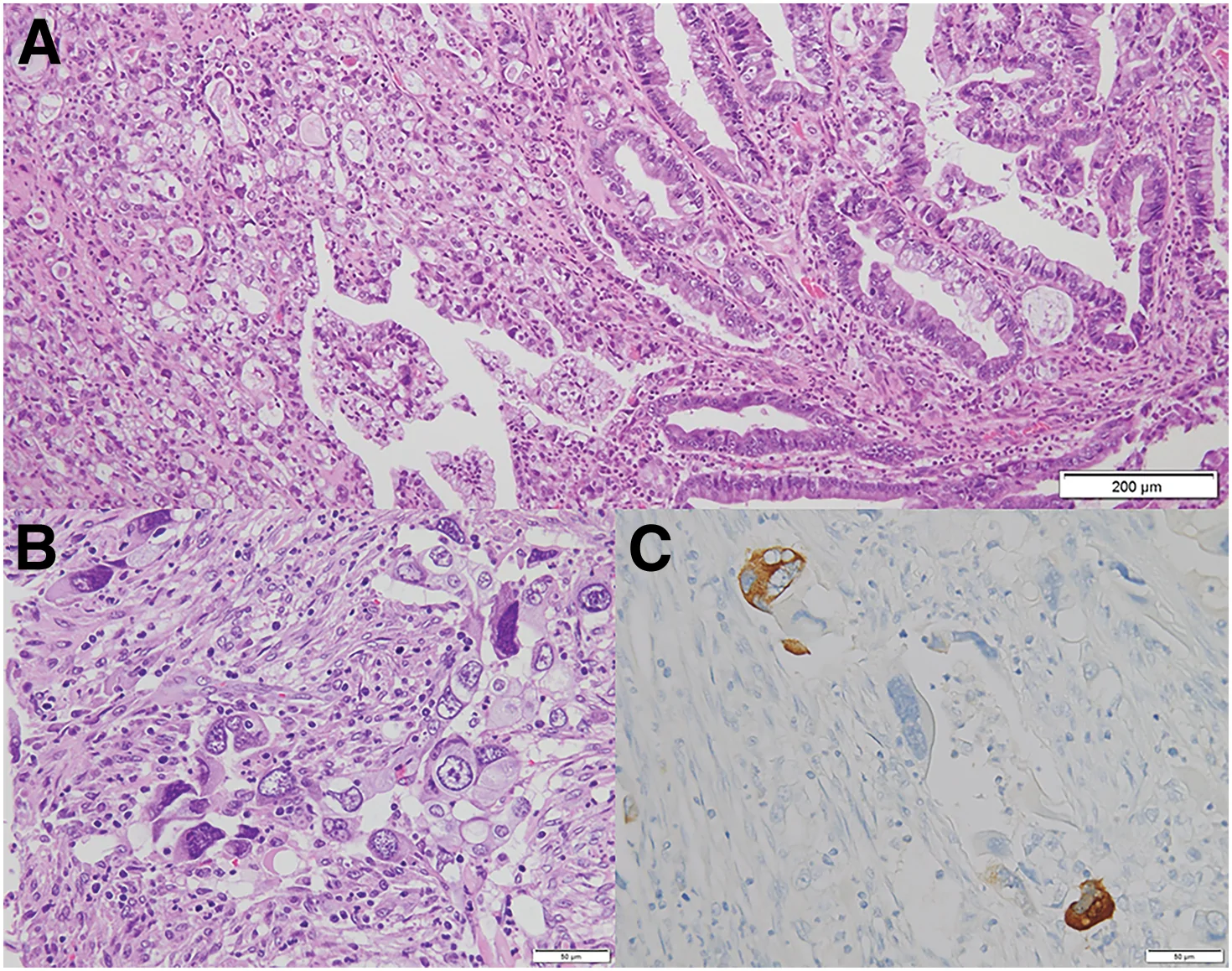
(**A**) Histological examination demonstrated predominantly well-differentiated adenocarcinoma (right) with an admixed poorly differentiated component (left). (**B**) Higher-magnification view of the poorly differentiated adenocarcinoma component, showing tumor cells with enlarged, irregular, hyperchromatic nuclei. (**C**) Immunohistochemical staining demonstrated HCG expression in a subset of tumor cells with enlarged, irregular, hyperchromatic nuclei. HCG human chorionic gonadotropin

The pathological stage was ypT3, ypN2, M0, Stage IVB according to the eighth edition of the Union for International Cancer Control (UICC) TNM classification of malignant tumors,^7)^ and R0 resection was achieved. The tumor directly invaded the adjacent liver and duodenum. The tumor consisted predominantly of well-differentiated adenocarcinoma, with areas showing varying degrees of differentiation. Poorly differentiated tumor cells with enlarged, irregular, hyperchromatic nuclei were occasionally observed (**Fig. 3A**). Immunohistochemical staining demonstrated focal positivity for HCG in scattered tumor cells (**Fig. 3B**). HCG immunoreactivity was observed as diffuse and granular cytoplasmic staining in a subset of tumor cells, including some poorly differentiated tumor cells. However, the distribution of HCG-positive cells did not completely overlap with that of the poorly differentiated component. Because the tumor morphology was not consistent with a malignant neoplasm composed of atypical trophoblastic cells, the tumor did not meet the diagnostic criteria for CC. The tumor was finally diagnosed as gallbladder adenocarcinoma with HCG-positive tumor cells. The pathological response to chemotherapy was classified as grade 1a according to the Evans grading system.

The patient received S-1 as adjuvant chemotherapy. However, local recurrence was detected 9 months after radical surgery. Because the response to preoperative GCD therapy was poor both radiologically and pathologically, GCP therapy was initiated. However, obstructive jaundice and cholangitis developed because of a malignant anastomotic stricture. External and internal drainage via PTBD was performed, and chemotherapy was continued. Despite these treatments, the patient eventually died 20 months after the initial diagnosis.

## DISCUSSION

HCG-associated GBC is extremely rare. To date, only a limited number of cases have been reported in the literature. **Table 1** summarizes cases of HCG-associated GBC.^2–6,8–11)^ HCG-associated GBC can be histologically classified into 3 groups: (1) conventional gallbladder adenocarcinoma with HCG-positive tumor cells^5)^; (2) CC^2,3,6,10)^; and (3) other rare histological subtypes, such as ASC and undifferentiated carcinoma.^4,8,9)^ Even in cases diagnosed as CC, ASC, or undifferentiated carcinoma, an adenocarcinoma component was frequently identified within the same tumor. These tumors may also be stratified according to the presence of clinically detectable HCG production. While many reports have documented elevated serum or urine HCG levels indicative of true HCG production,^2,4,6,8,9)^ such measurements were not performed in some previously reported cases. Consequently, a definitive distinction between HCG-producing tumors and those with HCG expression detected only by immunohistochemistry cannot be established. Therefore, we used the term HCG-associated GBC to collectively describe gallbladder tumors with documented HCG expression confirmed by immunohistochemistry and/or HCG production. In the current case, HCG immunoreactivity was confirmed in adenocarcinoma cells. Although poorly differentiated components were identified, the tumor did not meet the morphological criteria for CC and was therefore diagnosed as gallbladder adenocarcinoma with HCG-positive tumor cells. Because the pathological diagnosis of HCG-positive GBC was established approximately 2 weeks after surgery, serum and urine HCG levels were not measured perioperatively or at the time of recurrence.

**Table 1:** Summary of 17 HCG-associated GBC cases published in the English-language literature, including our case

No.	Ref.	Year	Author	Histology	Age	Sex	Distant metastases	Treatment	Regimen	Prognosis
1	^10)^	1981	Albores-Saavedra	CC	63	F	N/A	None*	–	N/A
2	^8)^	1988	Guo	UC	68	F	N/A	Surgery	–	Died (3 months)
3	^8)^	1988	Guo	UC	65	F	N/A	Surgery	–	Died (8 months)
4	^8)^	1988	Guo	UC	68	F	N/A	Chemotherapy*	N/A	Died (N/A)
5	^8)^	1988	Guo	UC	68	F	N/A	Chemotherapy*	N/A	Died (N/A)
6	^8)^	1988	Guo	UC	69	M	N/A	Surgery	–	Died (3 months)
7	^8)^	1988	Guo	UC	61	M	N/A	Surgery	–	Died (3 months)
8	^8)^	1988	Guo	UC	66	M	N/A	Surgery	–	Alive (19 months)
9	^8)^	1988	Guo	UC	61	F	N/A	Chemotherapy*	N/A	Died (N/A)
10	^8)^	1988	Guo	UC	62	F	N/A	Chemotherapy*	N/A	Died (N/A)
11	^9)^	1990	Fukuda	ASC	83	F	Peritoneum	Tumor resection	None	Died (N/A)
12	^2)^	1991	Abu-Farsakh	CC	29	F	Liver	Cholecystectomy	MTX	Died (1 month)
13	^5)^	2010	Sato	AC	79	M	None	Cholecystectomy	None	Alive (19 months)
14	^3)^	2001	Wang	CC	48	F		Cholecystectomy →Chemotherapy	EP	Rec (5 months) Died (12 months)
15	^4)^	2018	Leostic	UC	31	F	None	Hepatectomy**	MTX	Rec (few weeks)
16	^6)^	2023	Wang	CC	58	F	Liver	Chemotherapy →Cholecystectomy	GCC	Alive (19 months)
17		2026	Ours	AC	84	F	None	Chemotherapy →Hepatectomy** →Chemotherapy	GCD, GCP	Rec (9 months) Died (20 months)

*Autopsy cases.

**Cholecystectomy with a neighborhood hepatectomy.

AC, adenocarcinoma; ASC, adenosquamous carcinoma; CC, choriocarcinoma; EP, etoposide and cisplatin; F, female; GBC, gallbladder cancer; GCC, gemcitabine, cisplatin, and camrelizumab; GCD, gemcitabine, cisplatin, and durvalumab; GCP, gemcitabine, cisplatin, and pembrolizumab; HCG, human chorionic gonadotropin; M, male; MTX, methotrexate; N/A, not applicable; UC, undifferentiated carcinoma

Extragonadal CC is a malignant tumor arising outside the gonads that is characterized by the proliferation of atypical trophoblastic cells, including cytotrophoblasts and syncytiotrophoblasts.^11,12)^ CCs are generally characterized by HCG expression.^12)^ Although CCs arising outside the gonads are rare, several reports of primary gastrointestinal CCs, particularly in the stomach, have been published.^13,14)^ Two hypotheses have been proposed to explain this phenomenon: aberrant migration of ectopic germ cells or totipotent cells, and dedifferentiation from adenocarcinoma.^13)^ The dedifferentiation hypothesis suggests that malignant adenocarcinoma tissue dedifferentiates to a level resembling embryonal ectoderm, thereby retaining the potential to form trophoblastic cells.^12)^ This hypothesis is supported by the observation that gastrointestinal CCs occasionally exhibit a gradual transition from adenocarcinoma to CC.

The origin of HCG-positive, non-choriocarcinomatous tumor cells may also represent metaplastic differentiation or dedifferentiation. In our literature review, all reported cases of gallbladder CC consisted of a mixture of adenocarcinoma and CC components. Undifferentiated cases of HCG-positive GBC have also been reported.^4,8)^ HCG immunoreactivity has often been identified in gallbladder adenocarcinoma, particularly in poorly differentiated areas.^5)^ These findings suggest that gallbladder adenocarcinoma may dedifferentiate and acquire the ability to express HCG. In the current case, histopathological examination revealed, in addition to conventional well-differentiated adenocarcinoma, a population of tumor cells with marked nuclear atypia, characterized by enlarged and irregular nuclei. Based on these findings, immunohistochemical staining for HCG was performed. Immunohistochemical examination demonstrated HCG expression corresponding predominantly to these atypical tumor cells. However, the tumor was diagnosed as gallbladder adenocarcinoma with focal HCG expression, because the morphological features required for a diagnosis of CC were absent. The HCG expression was predominantly observed in poorly differentiated areas, which is consistent with previous reports.

The clinical features of HCG-associated GBC are similar to those of primary gallbladder adenocarcinoma, except for elevated serum or urine HCG levels in HCG-producing GBC. Many reported resected cases have been treated surgically according to treatment strategies for primary GBC.^2–5,9)^ Extended cholecystectomy, sometimes combined with hepatectomy and/or lymphadenectomy, is indicated for locally limited disease.^2–5)^ In contrast, chemotherapy is administered for metastatic or recurrent cases.^6)^

The outcomes of chemotherapy for non-gestational CC remain unsatisfactory.^13)^ Chemotherapy regimens that have been successfully used to treat gestational CC appear to be ineffective.^3)^ Because all reported cases of gallbladder CC have consisted of a mixture of adenocarcinoma and CC components, gemcitabine-based chemotherapy is indicated according to primary GBC treatment guidelines.^15)^ Because 1 patient treated with gemcitabine and cisplatin plus an ICI achieved complete remission, ICIs may achieve a significant anticancer effect in certain cases.^6)^ In the current case, GCD therapy was administered preoperatively for locally advanced GBC. However, the radiological and pathological treatment responses were poor. Although S-1 was administered as adjuvant chemotherapy, the tumor recurred shortly thereafter. After recurrence, GCP therapy was initiated because GCD therapy had been ineffective preoperatively. Chemotherapy, including ICIs, was ineffective, and the patient died 20 months after the initial diagnosis.

## CONCLUSIONS

We report a case of HCG-positive GBC. Although surgical treatment may be considered in resectable cases, the disease is generally chemoresistant and associated with a poor prognosis.

## DECLARATIONS

### Availability of data and materials

All data generated or analyzed during this study are included in this published article.
